# Groupwise image registration based on a total correlation dissimilarity measure for quantitative MRI and dynamic imaging data

**DOI:** 10.1038/s41598-018-31474-7

**Published:** 2018-08-30

**Authors:** Jean-Marie Guyader, Wyke Huizinga, Dirk H. J. Poot, Matthijs van Kranenburg, André Uitterdijk, Wiro J. Niessen, Stefan Klein

**Affiliations:** 1 Biomedical Imaging Group Rotterdam, Departments of Radiology and Medical Informatics, Erasmus MC - University Medical Centre Rotterdam, Rotterdam, The Netherlands; 2 Imaging Science and Technology, Faculty of Applied Sciences, Delft University of Technology, Delft, The Netherlands; 3 Departments of Radiology, Erasmus MC - University Medical Centre Rotterdam, Rotterdam, The Netherlands; 4 Department of Cardiology, Erasmus MC - University Medical Centre Rotterdam, Rotterdam, The Netherlands

## Abstract

The most widespread technique used to register sets of medical images consists of selecting one image as fixed reference, to which all remaining images are successively registered. This pairwise scheme requires one optimization procedure per pair of images to register. Pairwise mutual information is a common dissimilarity measure applied to a large variety of datasets. Alternative methods, called groupwise registrations, have been presented to register two or more images in a single optimization procedure, without the need of a reference image. Given the success of mutual information in pairwise registration, we adapt one of its multivariate versions, called total correlation, in a groupwise context. We justify the choice of total correlation among other multivariate versions of mutual information, and provide full implementation details. The resulting total correlation measure is remarkably close to measures previously proposed by Huizinga *et al*. based on principal component analysis. Our experiments, performed on five quantitative imaging datasets and on a dynamic CT imaging dataset, show that total correlation yields registration results that are comparable to Huizinga’s methods. Total correlation has the advantage of being theoretically justified, while the measures of Huizinga *et al*. were designed empirically. Additionally, total correlation offers an alternative to pairwise mutual information on quantitative imaging datasets.

## Introduction

Intensity-based image registration using the maximization of mutual information is commonly used for aligning pairs of medical images that do not have similar intensity distributions, or are acquired from different modalities^1–3^. Mutual information belongs to the family of pairwise dissimilarity measures. Pairwise methods quantify the alignment of a moving image with a fixed reference image. The optimization process performed in the context of pairwise registration therefore considers only two images simultaneously.

Nowadays, imaging datasets often contain more than two images, acquired from different modalities, different time points or different subjects, for instance. When more than two images have to be registered, the pairwise registration scheme is not always the most adapted. Firstly, the choice of reference image to which the remaining image are registered can be arbitrary, but may also influence the registration results, as shown by Geng *et al*.^4^. Secondly, pairwise registration does not allow the registration of all images in a single optimization procedure, which prevents taking into account all image information simultaneously.

Conversely, groupwise image registration methods are fully symmetric (i.e. all images play the same role in the registration procedure), and they consist of a single optimization procedure. Given the success of mutual information in the context of pairwise image registration, this paper specifically focuses on groupwise registration techniques that are based on the concept of mutual information. Though the formulation of mutual information for two images is unique, several multivariate versions exist for its generalization for more than two images. We provide theory about the main multivariate dissimilarity measures based on mutual information, that could be used for the groupwise registration of medical images. These dissimilarity measures are called interaction information^5^, total correlation^6^ and dual total correlation^7^. Total correlation is the groupwise dissimilarity measure that we propose to adapt in the context of groupwise image registration.

A preliminary version of our work was presented at a conference^8^. In the present article, we provide full theoretical developments, extensive implementation details, and additional experimental analyses.

Competing state-of-the-art dissimilarity measures for groupwise registration include the sum of variances developed by Metz *et al*.^9^, the groupwise mutual information method of Bhatia *et al*.^10^, and the groupwise dissimilarity measures based on principal component analysis (PCA) previously developed by Huizinga *et al*.^11^. The expression of the total correlation dissimilarity measure that we propose is remarkably close to Huizinga’s PCA-based groupwise dissimilarity measures, which were shown to outperform competing pairwise and groupwise state-of-the-art methods on qMRI datasets. The experiments conducted in this article consist of using groupwise total correlation for the registration of a dynamic CT imaging dataset, and of five quantitative magnetic resonance imaging (qMRI) image datasets. Registration results are compared to Huizinga’s methods, but also to pairwise registration based on mutual information.

## Results

Groupwise registration based on the total correlation dissimilarity measure ${{\mathscr{D}}}_{{\rm{TC}}}$ that we propose in this study is tested on six different types of image datasets, which overall represents 42 subjects. Dynamic series of CT images were acquired for the first type of image dataset, denoted CT-LUNG. The five other types of datasets, denoted T1MOLLI-HEART, T1VFA-CAROTID, ADC-ABDOMEN, DTI-BRAIN, and DCE-ABDOMEN, are qMRI datasets for which multiple MR images were acquired using different acquisition parameters (or at multiple time points after injection of a contrast agent). For these five qMRI datasets, we fitted a qMRI model to the image intensities at each spatial location, and extracted quantitative images: spin-lattice relaxation time (*T*_1_) images for T1MOLLI-HEART and T1VFA-CAROTID, apparent diffusion coefficient (ADC) images for ADC-ABDOMEN, mean diffusivity (MD) images for DTI-BRAIN, and transfer constant (*K*^*trans*^) images for DCE-ABDOMEN. More details on the image datasets are provided in the Experiments section of the present article.

### Registration accuracy

**Figure 1:**
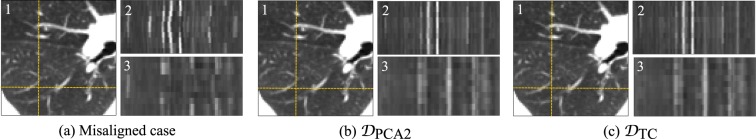
Registration results for a CT-LUNG dataset. The images denoted ‘2’ and ‘3’ stack the voxel information of *G* = 10 images at the locations defined by the dotted lines drawn in the image denoted ‘1’ (vertical line: ‘2’, horizontal line: ‘3’).

Figure 1 provides a visualization of the image alignment for a CT-LUNG dataset, gathering 10 CT images acquired at different time points from the lung area of a patient. Misalignments due to respiratory motion are visible when no registration is applied between the images (Fig. 1a), while they disappear after applying image registration based on Huizinga’s ${{\mathscr{D}}}_{{\rm{PCA2}}}$ (Fig. 1b) or on the total correlation dissimilarity measure ${{\mathscr{D}}}_{{\rm{TC}}}$ proposed in this article (Fig. 1c). Visual differences between the results obtained with ${{\mathscr{D}}}_{{\rm{PCA2}}}$ and ${{\mathscr{D}}}_{{\rm{TC}}}$ are more limited and harder to identify.

**Figure 2:**
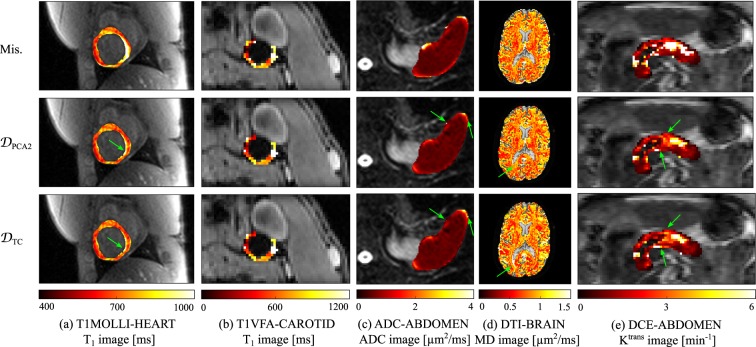
Tissue maps generated before image registration (top), after image registration with ${{\mathscr{D}}}_{{\rm{PCA2}}}$ (middle), and after image registration with ${{\mathscr{D}}}_{{\rm{TC}}}$ (bottom). The fitted values are shown in the myocardium for T1MOLLI-HEART, in the carotid artery wall for T1VFA-ABDOMEN, in the spleen for ADC-ABDOMEN, in the brain parenchyma for DTI-BRAIN, and in the pancreas for DCE-ABDOMEN. Slight visual changes between the tissue maps obtained with ${{\mathscr{D}}}_{{\rm{PCA2}}}$ and ${{\mathscr{D}}}_{{\rm{TC}}}$ are identified by green arrows.

For the five qMRI datasets, Fig. 2 provides quantitative parameter images obtained by applying curve fitting to the images before registration, after registration using Huizinga’s ${{\mathscr{D}}}_{{\rm{PCA2}}}$ groupwise dissimilarity measure, and after registration using the total correlation dissimilarity measure ${{\mathscr{D}}}_{{\rm{TC}}}$ proposed in this article. The fitting models used to derive the qMRI images assume that spatial correspondence is ensured between the images used for curve fitting. It is therefore expected that quantitative images obtained after image registration will be more reliable than without image registration^11,12^. Based on Fig. 2, visual differences in the estimates tissue maps are easily noticeable between the case before image registration, on the one hand, and the cases with ${{\mathscr{D}}}_{{\rm{PCA2}}}$ or ${{\mathscr{D}}}_{{\rm{TC}}}$, on the other hand. Such differences are particularly visible at organ interfaces. Slighter changes, identified by green arrows, can be identified between the tissue maps obtained with ${{\mathscr{D}}}_{{\rm{PCA2}}}$ and ${{\mathscr{D}}}_{{\rm{TC}}}$.

Full registration accuracy results in terms of landmark/volume correspondence (mTRE or Dice coefficient), registration transformation smoothness (denoted ${{\rm{STD}}}_{{\rm{\det }}(\partial {{\boldsymbol{T}}}_{{g}}/\partial {\bf{x}})}$), and uncertainty estimation (Cramér-Rao lower bound, denoted CRLB), are provided as supplementary material (Tables S1 to S6) for the following dissimilarity measures: pairwise mutual information ${{\mathscr{D}}}_{{\rm{MI}}}$, Huizinga’s dissimilarity measures based on PCA ${{\mathscr{D}}}_{{\rm{PCA}}}$ and ${{\mathscr{D}}}_{{\rm{PCA2}}}$, and the total correlation dissimilarity measure proposed in this article ${{\mathscr{D}}}_{{\rm{TC}}}$.

Table 1 presents a partial version of the registration accuracy results, based on the middle value of the control point spacings that were used for the non-rigid B-spline transformation model: 13 mm for CT-LUNG, 64 mm for T1MOLLI-HEART, 16 mm for T1VFA-CAROTID, 64 mm for ADC-ABDOMEN, and 64 mm for DCE-ABDOMEN. Registration performances in terms of landmark correspondence (mean target registration error, denoted mTRE) or overlap of volumes of interest (Dice coefficients) are given in Table 1a. For all dataset, better alignments (i.e. lower mTRE) or overlaps (i.e. higher Dice coefficients) were obtained with the groupwise measures ${{\mathscr{D}}}_{{\rm{TC}}}$, ${{\mathscr{D}}}_{{\rm{PCA}}}$ and ${{\mathscr{D}}}_{{\rm{PCA2}}}$ than with pairwise mutual information ${{\mathscr{D}}}_{{\rm{MI}}}$, with one exception: the mTRE obtained with ${{\mathscr{D}}}_{{\rm{PCA2}}}$ for the CT-LUNG dataset is higher than the mTRE obtained with ${{\mathscr{D}}}_{{\rm{MI}}}$. The Dice coefficients and mTRE results are very similar for ${{\mathscr{D}}}_{{\rm{TC}}}$, ${{\mathscr{D}}}_{{\rm{PCA}}}$ and ${{\mathscr{D}}}_{{\rm{PCA2}}}$. The only case for which ${{\mathscr{D}}}_{{\rm{TC}}}$ performs slightly worse than the two other groupwise measures is on the DCE-ABDOMEN dataset. Table 1b provides values for the transformation smoothness ${{\rm{STD}}}_{{\rm{\det }}(\partial {{\bf{T}}}_{g}/\partial {\bf{x}})}$. In all cases, ${{\mathscr{D}}}_{{\rm{TC}}}$, ${{\mathscr{D}}}_{{\rm{PCA}}}$ and ${{\mathscr{D}}}_{{\rm{PCA2}}}$ yield lower (i.e. better) values of ${{\rm{STD}}}_{{\rm{\det }}(\partial {{\boldsymbol{T}}}_{g}/\partial {\bf{x}})}$ than ${{\mathscr{D}}}_{{\rm{MI}}}$. The only case for which ${{\mathscr{D}}}_{{\rm{TC}}}$ performs slightly worse than the two other groupwise measures is on the T1VFA-CAROTID dataset. Table 1c provides uncertainty estimations of the qMRI fit (90^th^ $\sqrt{{\rm{CRLB}}}$). The results indicate that the values of 90^th^ $\sqrt{{\rm{CRLB}}}$ are lower (i.e. better) with ${{\mathscr{D}}}_{{\rm{TC}}}$ than with ${{\mathscr{D}}}_{{\rm{MI}}}$ for the T1MOLLI-HEART and DCE-ABDOMEN datasets, while they are quite similar for T1VFA-CAROTID and DTI-BRAIN, and higher (i.e. worse) for the ADC-ABDOMEN dataset. The 90^th^ $\sqrt{{\rm{CRLB}}}$ obtained with ${{\mathscr{D}}}_{{\rm{TC}}}$ is higher than the 90^th^ $\sqrt{{\rm{CRLB}}}$ obtained with ${{\mathscr{D}}}_{{\rm{PCA}}}$ and ${{\mathscr{D}}}_{{\rm{PCA2}}}$ for two datasets (ADC-ABDOMEN and DCE-ABDOMEN), while it is similar or better for three datasets (T1MOLLI-HEART, T1VFA-CAROTID, and DTI-BRAIN). The full results (Tables S1 to S6) are consistent with the results presented in Table 1a–c.

**Table 1:** Registration results.

	CT	T1MOLLI	T1VFA	ADC	DTI	DCE
	LUNG	HEART	CAROTID	ABDOMEN	BRAIN	ABDOMEN
**(a) Dice coefficients or mTRE values (mean value** ± **standard deviation)**						
	***mTRE*** [**mm**]	***Dice*** [%]	***mTRE*** [**mm**]	***Dice*** [%]	—	***mTRE*** [**mm**]
Mis.	6.72 ± 2.51	48 ± 8	1.47 ± 0.54	70 ± 4	—	8.49 ± 4.54
${{\mathscr{D}}}_{{\rm{MI}}}$	1.43 ± 0.23	37 ± 11	1.22±0.43	64 ± 16	—	6.46 ± 2.32
${{\mathscr{D}}}_{{\rm{PCA}}}$	1.40 ± 0.37	53 ± 7	1.11 ± 0.42	71 ± 5	—	6.11 ± 2.33
${{\mathscr{D}}}_{{\rm{PCA2}}}$	1.56 ± 0.55	52 ± 11	1.08 ± 0.39	75 ± 5	—	5.99 ± 2.18
${{\mathscr{D}}}_{{\rm{TC}}}$	1.42 ± 0.40	53 ± 11	1.09 ± 0.40	74 ± 5	—	6.18 ± 2.40
**(b) Transformation smoothness** ${{\rm{STD}}}_{{\rm{\det }}(\partial {{\boldsymbol{T}}}_{g}/\partial {\bf{x}})}$ **[%] (mean value ± standard deviation)**						
Mis.	0 ± 0	0 ± 0	0 ± 0	0 ± 0	—	0 ± 0
${{\mathscr{D}}}_{{\rm{MI}}}$	15 ± 4	7 ± 2	2 ± 0	8 ± 3	—	4 ± 2
${{\mathscr{D}}}_{{\rm{PCA}}}$	8 ± 2	2 ± 1	2 ± 1	3 ± 2	—	4 ± 2
${{\mathscr{D}}}_{{\rm{PCA2}}}$	7 ± 2	1 ± 1	1 ± 0	3 ± 1	—	2 ± 1
${{\mathscr{D}}}_{{\rm{TC}}}$	8 ± 2	2 ± 1	1 ± 0	5 ± 2	—	4 ± 2
**(c) Uncertainty estimation 90** ^**th**^ $\sqrt{{\rm{CRLB}}}$ **of the fitted parameters (mean value ± standard deviation)**						
	**—**	***T***_**1**_ **[ms]**	***T***_**1**_ **[ms]**	***ADC*** **[** ***μm*** ^**2**^ **/ms]**	***MD*** **[** ***μm*** ^**2**^ **/ms]**	***K*** ^***trans***^ **[min** ^**−1**^ **]**
Mis.	—	92 ± 19	>1000	1.37 ± 0.83	0.096 ± 0.029	2.84 ± 2.30
${{\mathscr{D}}}_{{\rm{MI}}}$	—	97 ± 16	501 ± 83	0.25 ± 0.05	0.084 ± 0.028	3.64 ± 4.13
${{\mathscr{D}}}_{{\rm{PCA}}}$	—	87 ± 16	498 ± 93	0.23 ± 0.06	0.085 ± 0.029	1.52 ± 1.18
${{\mathscr{D}}}_{{\rm{PCA2}}}$	—	83 ± 12	510 ± 110	0.27 ± 0.05	0.084 ± 0.028	1.27 ± 0.92
${{\mathscr{D}}}_{{\rm{TC}}}$	—	77 ± 13	500 ± 96	0.32 ± 0.05	0.085 ± 0.028	1.87 ± 1.79

### Multivariate joint normality

**Figure 3:**
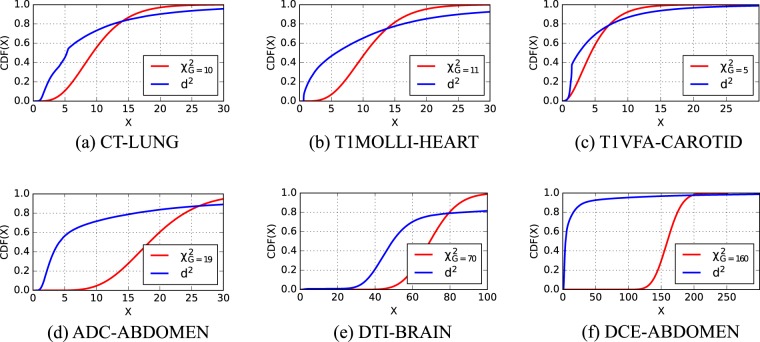
Cumulative distribution functions for one subject of the six image datasets (aligned case). The observed CDF (blue) is compared with the theoretical CDF of a chi-square distribution with G degrees of freedom (red).

As detailed in the Method section, the computation of the total correlation dissimilarity measure ${{\mathscr{D}}}_{{\rm{TC}}}$ that we propose is based on the approximation that the intensity distribution of the images to register is multivariate normal. Cumulative distribution functions (CDF) of the squared Mahalanobis distance *d*^2^, representing the intensity distribution for each of the six dataset types, are plotted in Fig. 3. According to these plots, none of these measure CDF follows the theoretical multivariate normal CDF (${\chi }_{G}^{2}$ distribution), which suggests that the image intensities of the images do not follow a multivariate normal distribution.

### Computational efficiency of total correlation ${{\mathscr{D}}}_{{\rm{TC}}}$

**Figure 4:**
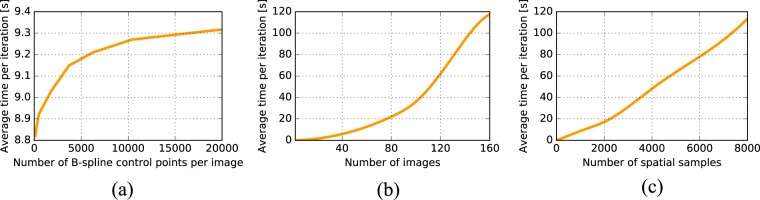
Average time per iteration with respect to the number of B-spline control points per image (**a**), the number of images *G* (**b**), and the number of spatial samples (**c**).

Figure 4 illustrates the evolution of the average time per iteration obtained with groupwise total correlation ${{\mathscr{D}}}_{{\rm{TC}}}$ for three image registration parameters: the number of B-spline control points per image, the number of images *G*, and the number of spatial samples taken to evaluate the dissimilarity measure. The results show that the average registration time per iteration monotonically increases with each of the considered registration parameter. With the present implementation of ${{\mathscr{D}}}_{{\rm{TC}}}$ and of the registration components of the elastix software used to perform the registrations, the results indicate that the number of B-spline control points has a limited influence on the average time per iteration as it remains close to 9 seconds for the whole span of numbers of B-spline control points that we considered. The experiments suggest that the number of images *G* influences the computation time most. For instance, when the number of image is *G* = 40, the average iteration time is 5 seconds, while this time reaches about two minutes for *G* = 160 images. In terms of the number of spatial samples, multiplying the number of spatial samples by 4 ends up in an average time per iteration that is multiplied by 6.

## Discussion

The focus of this paper was to adapt a multivariate version of mutual information in the context of the groupwise registration of medical images, so that it can be used to register two or more images in one optimization procedure.

Among the main multivariate versions of mutual information, namely interaction information ${{\mathscr{D}}}_{{\rm{II}}}$, total correlation ${{\mathscr{D}}}_{{\rm{TC}}}$ and dual total correlation ${{\mathscr{D}}}_{{\rm{TC}}}$, total correlation ${{\mathscr{D}}}_{{\rm{TC}}}$ theoretically allows to quantify the shared information between any subset of the images to register. Besides, the expression of total correlation is particularly straightforward to apply for the registration of *G* ≥ 2 images, provided that the image intensity distribution is approximated by a multivariate normal distribution.

The expression of the approximated total correlation dissimilarity measure ${{\mathscr{D}}}_{{\rm{TC}}}$ that we devise is remarkably analogous to the expressions of two other dissimilarity measures ${{\mathscr{D}}}_{{\rm{PCA}}}$ and ${{\mathscr{D}}}_{{\rm{PCA2}}}$ introduced by Huizinga *et al*.^11^, which were developed based on the intuition that an aligned set of images can be described by a small number of high eigenvalues. The expressions of these dissimilarity measures are all sums of functions of the eigenvalues of the correlation matrix **K** (compare Equations (18), (25) and (26)). Huizinga *et al*.^11^ had proposed to weigh more the last eigenvalues (the *λ*_*i*_ with the highest *i* indexes) than the first ones (the *λ*_*i*_ with the lowest *i* indexes) so that as much variance as possible is explained by a few large eigenvectors. The form of ${{\mathscr{D}}}_{{\rm{TC}}}$ obtained in this study confirms the intuition of Huizinga *et al*.^11^, since the natural logarithm in Equation (18) also puts more weight on the lower eigenvalues than on the higher ones.

Results obtained on a dynamic imaging dataset and on five multi-parametric datasets show that the total correlation method that we propose yields comparable results as PCA-based methods of Huizinga *et al*.^11^, and better registration results than pairwise mutual information ${{\mathscr{D}}}_{{\rm{MI}}}$. The main advantage of ${{\mathscr{D}}}_{{\rm{TC}}}$ with respect to ${{\mathscr{D}}}_{{\rm{PCA}}}$ and ${{\mathscr{D}}}_{{\rm{PCA2}}}$ is that it is more theoretically justified: the contribution of each eigenvalue used to compute ${{\mathscr{D}}}_{{\rm{TC}}}$ is automatically calibrated and follows naturally from the concepts of multivariate mutual information, whereas for ${{\mathscr{D}}}_{{\rm{PCA}}}$ and ${{\mathscr{D}}}_{{\rm{PCA2}}}$, the eigenvalue calibration was empirically chosen.

Our study shows that even though the intensity distribution of the datasets to register is not multivariate normal (Fig. 3), ${{\mathscr{D}}}_{{\rm{TC}}}$ yields registration results that are better than mutual information and similar to the PCA dissimilarity measures of Huizinga *et al*.^11^. This is the case for a total of six diverse multi-parametric datasets, which suggests that approximating the intensity distributions, as done in this article, yields optimization minima that result in comparable or better registration accuracies than other state-of-the-art pairwise and groupwise techniques. On multi-parametric datasets, the results suggest that the approximation by a multivariate normal distribution is not detrimental to the registration results.

In the current implementation of the total correlation registration technique, increases in the number of images *G* have the largest impact on the average time per iteration, which is not surprising as both the amount of image data to register and the number of transformations to estimate scale with a factor *G*; moreover, estimating the correlation matrix **K** and its eigenvalue decomposition become increasingly computationally demanding. Further optimizations could improve the scalability of total correlation with respect to the number of images. The computation time also scales linearly with the number of spatial samples. Thanks to the use of the stochastic gradient descent optimization routine, we were able to use a relatively low number (2048) of spatial samples in our experiments, while still achieving accurate registration.

Other possible applications of the total correlation dissimilarity measure proposed in this article include motion tracking in ultrasound image sequences^13,14^, motion compensation in dynamic PET^15^ or dynamic contrast-enhanced CT^16^, and for population template construction^17^. Future research should validate the performance of the method in such contexts.

## Conclusion

In conclusion, we proposed an implementation of an approximated version of total correlation ${{\mathscr{D}}}_{{\rm{TC}}}$ for which the registration results are comparable to the results obtained with the dissimilarity measures of Huizinga *et al*.^11^, on multi-parametric datasets. Our results indicate that approximating the intensity distributions by a joint normal distribution for the sake of efficient calculation of the entropy, used to derive total correlation ${{\mathscr{D}}}_{{\rm{TC}}}$, does not constitute a limitation in the practical application of ${{\mathscr{D}}}_{{\rm{TC}}}$ to quantitative imaging datasets. Total correlation ${{\mathscr{D}}}_{{\rm{TC}}}$ has the advantage of being elegant and theoretically justified, while the dissimilarity measures ${{\mathscr{D}}}_{{\rm{PCA}}}$ and ${{\mathscr{D}}}_{{\rm{PCA2}}}$ proposed by Huizinga *et al*.^11^ were elaborated empirically. Additionally, groupwise total correlation ${{\mathscr{D}}}_{{\rm{TC}}}$ offers an alternative to pairwise registration based on mutual information on multi-parametric imaging datasets.

## Method

Let us consider ${\mathcal M} =\{{M}_{1},\,\mathrm{...,}\,{M}_{G}\}$, a series of *G* images that have to be registered. Each image *M*_*g*_, consists of *N* voxels. To quantify how well the *G* images are aligned, a dissimilarity measure has to be defined. In this study, we consider dissimilarity measures based on the concepts of mutual information. We choose the convention to formulate the measures as dissimilarity measures instead of similarity measures, so that the registration problem can be written as a cost function minimization problem.

### Pairwise mutual information

Mutual information is a robust measure that is commonly used for the pairwise registration of datasets of medical images, including multimodal datasets^3^. For *G* = 2 images *M*_1_ and *M*_2_, the negated mutual information ${{\mathscr{D}}}_{{\rm{MI}}}$ is computed as follows^1,3^:

1 $${{\mathscr{D}}}_{{\rm{MI}}}({M}_{1},\,{M}_{2})=H({M}_{1},{M}_{2})-H({M}_{1})-H({M}_{2})$$

with *H*(*M*_1_) the entropy^18^ of image *M*_1_, *H*(*M*_2_) the entropy of image *M*_2_, and *H*(*M*_1_, *M*_2_) the joint entropy of *M*_1_ and *M*_2_. For two images *M*_1_ and *M*_2_, the joint entropy can be computed as follows^19^:

2 $$H({M}_{1},\,{M}_{2})=-\sum _{{x}_{1}}\sum _{{x}_{2}}\,P({x}_{1},\,{x}_{2})\,\mathrm{ln}\,[P({x}_{1},\,{x}_{2})]$$

where *x*_1_ and *x*_2_ represent the discrete values of images *M*_1_ and *M*_2_, respectively. *P*(*x*_1_, *x*_2_) is the probability of these values occuring together. *P*(*x*_1_, *x*_2_) ln[*P*(*x*_1_, *x*_2_)] is defined to be 0 if *P*(*x*_1_, *x*_2_) equals 0.

**Figure 5:**
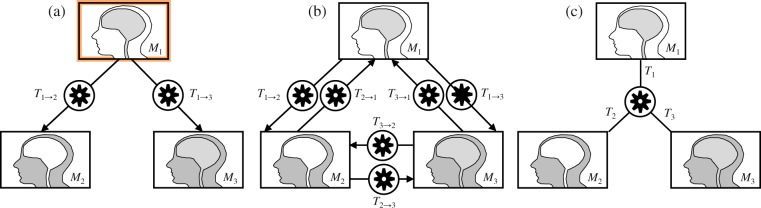
(**a**) Pairwise registration scheme (the orange frame indicates that this method requires the selection of a reference image), (**b**) semi-groupwise registration scheme proposed by Seghers *et al*.^20^, and (**c**) groupwise registration scheme.

When the dataset of images to register contains *G* > 2 images, it is still possible to use a pairwise method to register the images, but several independent registration procedures have to be performed. A typical method consists of selecting one of the images as fixed reference, and then successively applying pairwise registration with the remaining *G*−1 images considered as moving images (Fig. 5a). This technique is not well suited to registration problems for which there is no obvious reference image. Besides, the registration results may be different according to the choice of fixed reference image, as shown by Geng *et al*.^4^. Seghers *et al*.^20^ introduced a method that we will refer to as semi-groupwise, which is based on multiple pairwise registrations and does not require the selection of a reference space. For each *i*, *M*_*i*_ is taken as fixed image and *G*−1 independent registration are performed between each remaining image, *M*_*j*_, yielding *G*−1 transformations *T*_*i*→*j*_ per fixed image *M*_*i*_. Each image *M*_*i*_ is then resampled into an average or mid-point image space using ${\bar{T}}_{i}^{-1}$(***x***), the inverse of the arithmetic mean of the transformations *T*_*i*→*j*_ (Fig. 5b). The method of Seghers *et al*.^20^ has the disadvantage of requiring *G* × (*G*−1) registration procedures, which becomes computationally complex when *G* grows. It also does not allow to register all images in a single optimization procedure.

### Groupwise dissimilarity measures based on multivariate mutual information

Groupwise registration techniques allow to register *G* ≥ 2 images. In this study, we will focus on groupwise techniques that allow to register all images in one optimization procedure, and that treat the images equally (Fig. 5c). In particular, the order in which the images are supplied should have no influence on the value of the groupwise dissimilarity measure ${\mathscr{D}}({M}_{1},\,{M}_{2},\,\mathrm{...,}\,{M}_{G})$, and therefore no influence on the registration results.

This article more precisely focuses on groupwise generalizations of mutual information, given the wide interest and range of applications of that dissimilarity measure in the context of pairwise image registration^3^. There exist multiple multivariate forms of mutual information^5–7^, the concepts of which can be used for groupwise image registration.

The first multivariate generalization of mutual information is known as interaction information^5^, denoted ${{\mathscr{D}}}_{{\rm{II}}}$. It measures the amount of information shared by all the images. For the *G* images of ${\mathcal M}$, negated interaction information is written:

3 $${{\mathscr{D}}}_{{\rm{II}}}( {\mathcal M} )=\sum _{V\subseteq  {\mathcal M} }\,{(-\mathrm{1)}}^{G-|V|}H(V)$$

with $V\subseteq  {\mathcal M}$ meaning that *V* can be any subset of images of ${\mathcal M}$ (e.g. if *G* = 3, then *V* successively represents the following subsets of images: {*M*_1_}, {*M*_2_}, {*M*_3_}, {*M*_1_, *M*_2_}, {*M*_1_, *M*_3_}, {*M*_2_, *M*_3_}, and {*M*_1_, *M*_2_, *M*_3_}), |*V*| the number of images in the corresponding subset, and *H*(*V*) the joint entropy of the subset *V*. For *G* images *M*_1_...*M*_*G*_, the joint entropy is the generalization of Equation (2):

4 $$H({M}_{1},\,\mathrm{...,}\,{M}_{G})=-\sum _{{x}_{1}}\mathrm{...}\sum _{{x}_{G}}\,P({x}_{1},\,\mathrm{...,}\,{x}_{G})\,\mathrm{ln}\,[P({x}_{1},\,\mathrm{...,}\,{x}_{G})]$$

where the *x*_1_, ..., *x*_*G*_ are the values of images *M*_1_, ..., *M*_*G*_, respectively. The same definitions as for *P*(*x*_1_, *x*_2_) and *P*(*x*_1_, *x*_2_)ln[*P*(*x*_1_, *x*_2_)] are directly extended for *P*(*x*_1_, ..., *x*_*G*_) and *P*(*x*_1_,..., *x*_*G*_)ln[*P*(*x*_1_, ..., *x*_*G*_)]. Interaction information quantifies the information shared together by images *M*_1_, ..., *M*_*G*_^21^. This means that if at least one of the images of ${\mathcal M}$ shares no information with all other images, the interaction information will be zero^21,22^.

The second form of multivariate mutual information, called total correlation^6^, measures the amount of information shared between any subset of ${\mathcal M}$. The negated total correlation is written as:

5 $${{\mathscr{D}}}_{{\rm{TC}}}( {\mathcal M} )=H( {\mathcal M} )-[\sum _{g\mathrm{=1}}^{G}\,H({M}_{g})]$$

with $H( {\mathcal M} )$ the joint entropy of the images of the set ${\mathcal M} =\{{M}_{1},\,\mathrm{...,}\,{M}_{G}\}$.

The third form is a refinement of total correlation called dual total correlation^7^, and can be written as:

6 $${{\mathscr{D}}}_{{\rm{DTC}}}( {\mathcal M} )=[\sum _{g\mathrm{=1}}^{G}\,H({M}_{g}|( {\mathcal M} \backslash {M}_{g}))]-H( {\mathcal M} )$$

with ${\mathcal M} \backslash {M}_{g}$ the set of images {*M*_1_, ..., *M*_*G*_} without *M*_*g*_. $H({M}_{g}|( {\mathcal M} \backslash {M}_{g}))$ is the conditional entropy^19^ of *M*_*g*_ given ${\mathcal M} \backslash {M}_{g}$. In other terms, $H({M}_{g}|( {\mathcal M} \backslash {M}_{g}))$ is the entropy of the image *M*_*g*_ given the knowledge of images {*M*_1_, ..., *M*_*g*−1_, *M*_*g* +1_, ..., *M*_*G*_}.

**Figure 6:**
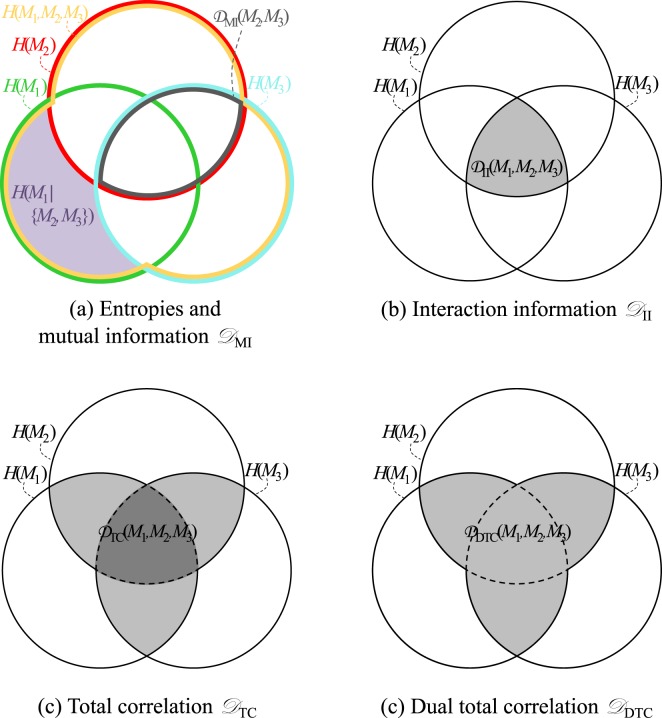
Venn diagram representations for three images *M*_1_, *M*_2_ and *M*_3_. (**a**) The green, red and cyan circle represent the entropy of each image. The fact that the images share information is symbolized by the fact that these circles overlap. Subfigures (**b**), (**c**) and (**d**) were constructed based on Equations (3), (5) and (6). In (**c**), the dark greay area signifies that its contribution to the dissimilarity measure is twice as high as the contribution of each light-grey area.

Theoretically, both total correlation and dual total correlation quantify the amount of shared information between all possible combinations of images, while interaction information only quantifies the amount of information shared by all images^23^. Venn diagrams^19,23,24^ for ${{\mathscr{D}}}_{{\rm{II}}}$, ${{\mathscr{D}}}_{{\rm{TC}}}$ and ${{\mathscr{D}}}_{{\rm{DTC}}}$ are shown in Fig. 6. In the context of image registration, ${{\mathscr{D}}}_{{\rm{TC}}}$ and ${{\mathscr{D}}}_{{\rm{DTC}}}$ seem more adapted than ${{\mathscr{D}}}_{{\rm{II}}}$, as they are built to quantify shared information not only between all images, but also between any of their subsets^21,22^. In particular, including an image with little dependence towards the others would impair the registration of the remaining images when using ${{\mathscr{D}}}_{{\rm{II}}}$, while this would theoretically not be the case when using ${{\mathscr{D}}}_{{\rm{TC}}}$ or ${{\mathscr{D}}}_{{\rm{DTC}}}$. We therefore chose to consider the dissimilarity measures based on total correlation in order to construct a groupwise dissimilarity measure.

### Groupwise total correlation

In this section, we describe how total correlation, as expressed in Formula (5), can be brought to practical use in the context of image registration. As such, computing total correlation implies computing the joint entropy $H( {\mathcal M} )$, but this computation is subject to the curse of dimensionality^25^: the evaluation of joint entropy requires to compute a *G*-dimensional joint histogram that becomes increasingly sparser as *G* increases, and therefore becomes computationally prohibitive.

Let us consider a random variable ${\bf{X}}\in {{\mathbb{R}}}^{G}$ following a *G*-variate normal distribution given by:

7 $$f({\bf{X}})=\frac{1}{\sqrt{{\rm{\det }}\mathrm{(2}\pi {\bf{C}})}}\exp (-\frac{1}{2}{({\bf{X}}-\mu )}^{{\rm{T}}}{{\bf{C}}}^{-1}({\bf{X}}-\mu ))$$

with $\mu \in {{\mathbb{R}}}^{G}$ an expectation vector, ${\bf{C}}\in {{\mathbb{R}}}^{G\times G}$ a covariance matrix, and with det(.) the determinant operator. Ali Ahmed *et al*.^26^ have shown that the entropy of the multivariate normal variable **X** may be written as:

8 $$H({\bf{X}})=\frac{G}{2}+\frac{G}{2}\,\mathrm{ln}\,\mathrm{(2}\pi )+\frac{1}{2}\,\mathrm{ln}\,({\rm{\det }}({\bf{C}}))$$

To circumvent the curse of dimensionality, and make it possible to use registration in a groupwise manner on datasets containing any number *G* ≥ 2 images, we propose to use Equation (8) in the context of *G* images ${\mathcal M} =\{{M}_{1},\,\mathrm{...,}\,{M}_{G}\}$. For the sake of efficient calculation of the entropy, we approximate the intensity distribution of the images by a joint normal distribution, and we make the hypothesis that the minimum of the resulting cost function is still a good solution for the underlying registration problem. Let **M** be a *N* × *G* matrix in which each image *M*_*g*_ is represented by a column. The matrix **C** of covariances between the images *M*_*g*_ is obtained as follows:

9 $${\bf{C}}=\frac{1}{N-1}{({\bf{M}}-\overline{{\bf{M}}})}^{{\rm{T}}}({\bf{M}}-\overline{{\bf{M}}})$$

with $\overline{{\bf{M}}}$, a matrix that has in each of its column the column-wise average of **M**. To make the method robust to linear intensity scalings and offsets, we incorporate an intensity standardization (i.e. z-score) within the definition of the dissimilarity measure. This is done by computing the entropy $H( {\mathcal M} )$ using the correlation matrix **K** instead of the covariance matrix **C**, with:

10 $${\bf{K}}={{\rm{\Sigma }}}^{-1}{\bf{C}}{{\rm{\Sigma }}}^{-1}$$

with Σ a diagonal matrix with the standard deviations of the columns of **M** as its diagonal elements. A diagonal element Σ_*gg*_ of Σ verifies:

11 $${{\rm{\Sigma }}}_{gg}=\frac{1}{N-1}\sum _{i\mathrm{=1}}^{N}\,{({M}_{g,i}-{\overline{M}}_{g})}^{2}$$

where the *M*_*g*,*i*_ are the individual voxel values and ${\overline{M}}_{g}$ the average voxel value of image ${\overline{M}}_{g}$. By construction, each diagonal element of the correlation matrix **K** is equal to 1. The expression of the joint entropy therefore becomes:

12 $$H( {\mathcal M} )=\frac{G}{2}+\frac{G}{2}\,\mathrm{ln}\,\mathrm{(2}\pi )+\frac{1}{2}\,\mathrm{ln}\,({\rm{\det }}({\bf{K}}))$$

Equation (12) can also be used to derive the marginal entropies *H*(*M*_*g*_). When considering only one image *M*_*g*_, the correlation matrix **K** is the scalar 1. All *H*(*M*_*g*_) are therefore constant and equal to:

13 $$H({M}_{g})=\frac{1}{2}+\frac{1}{2}\,\mathrm{ln}\,\mathrm{(2}\pi )$$

By combining Equations (5), (12) and (13), we define the dissimilarity measure based on total correlation ${{\mathscr{D}}}_{{\rm{TC}}}$ as follows:

14 $${{\mathscr{D}}}_{{\rm{TC}}}( {\mathcal M} )=\frac{1}{2}\,\mathrm{ln}({\rm{\det }}({\bf{K}})))=\frac{1}{2}\sum _{j=1}^{G}\,\mathrm{ln}\,{\lambda }_{j}$$

using ${\rm{\det }}({\bf{K}})={\prod }_{j=1}^{G}{\lambda }_{j}$, with *λ*_*j*_ the *j*^th^ eigenvalue of **K**, and *λ*_*j*_ > *λ*_*j*+1_. Such a simple expression was not found for dual total correlation, which is why we selected total correlation as groupwise dissimilarity measure.

### Gradient-based optimization and implementation

To implement the approximated version of ${{\mathscr{D}}}_{{\rm{TC}}}$ provided in Equation (14), we define an interpolation scheme based on B-splines. This scheme associates with each original image *M*_*g*_ a continuous and differentiable function *M*_*g*_(**x**) of the spatial coordinate **x**. The aim is to simultaneously bring the images *M*_*g*_(**x**) to an average space by means of a transformation ***T***(**x**, *μ*), where *μ* is a vector containing the parameters *μ*_*g*_ that correspond to the transformation ***T***_*g*_(**x**, *μ*_*g*_) related to each image *M*_*g*_. Examples of transformation models are the affine model, or the non-rigid model in which deformations are modeled by cubic B-splines^27^.

In the groupwise scheme, the measure ${\mathscr{D}}$ quantifies the dissimilarity between all transformed images *M*_*g*_(***T***_*g*_(**x**, *μ*_*g*_)). We adopted the pull-back definition of a warped image. Groupwise registration can therefore be formulated as the constrained minimization of the dissimilarity measure ${\mathscr{D}}$ with respect to *μ*, as previously proposed by Huizinga *et al*.^11^:

15 $$\hat{\mu }={\rm{\arg }}\,\mathop{{\rm{\min }}}\limits_{{\mu }}\,{\mathscr{D}}({M}_{1}({T}_{1}({\bf{x}},{{\mu }}_{1})),\,\mathrm{...,}\,{M}_{G}({T}_{G}({\bf{x}},\,{{\mu }}_{G})))$$

subject to the following constraint, allowing to define a mid-point space^28^:

16 $$\sum _{g=1}^{G}\,{{\mu }}_{g}=0$$

The implementation of the total correlation dissimilarity measure ${{\mathscr{D}}}_{{\rm{TC}}}$ was performed as part of the open source software package elastix^29^. The adaptive stochastic gradient descent (ASGD) developed by Klein *et al*.^30^ is used as optimization method for image registration. This method randomly samples positions in the image space at each iteration in order to reduce computation time. Sampling is done off the voxel grid, which was shown to be necessary to reduce interpolation artefacts^29^. A multi-resolution strategy is used: the images are Gaussian-blurred with a certain standard deviation, which is decreased at each resolution level. This means that the large deformations are corrected first, and that finer deformations are corrected in subsequent levels. Linear interpolation is used to interpolate the images during registration, which reduces computation time, but cubic B-spline interpolation was used to produce the final registered images. For the chosen ASGD optimization method, the gradient of the dissimilarity measure is needed. Based on Equation (14) and van der Aa *et al*.^31^, we find:

17 $$\frac{\partial {{\mathscr{D}}}_{{\rm{TC}}}}{\partial \mu }=\frac{1}{2}\sum _{j=1}^{G}\,\frac{1}{{\lambda }_{j}}\frac{\partial {\lambda }_{j}}{\partial {\mu }}=\frac{1}{2}\sum _{j=1}^{G}\,\frac{1}{{\lambda }_{j}}({{v}}_{j}^{{\rm{T}}}\frac{\partial {\bf{K}}}{\partial {\mu }}{{v}}_{j})$$

where ${v}_{j}^{T}$ is the *j*^th^ eigenvector of **K**. Similarly to van der Aa *et al*.^31^, we assume that the repetition of eigenvalues is unlikely.

When the eigenvalues *λ*_*j*_ tend towards zero, evaluating ${{\mathscr{D}}}_{{\rm{TC}}}$ implies taking the natural logarithm of a near-zero number (as shown in Equation (14)), which might result in a failing optimization. We therefore introduce an adjusting constant *c* ∈ ${{\mathbb{R}}}^{+}$ that is added to the eigenvalue *λ*_*j*_ before taking the natural logarithm:

18 $${{\mathscr{D}}}_{{\rm{T}}{\rm{C}}}({\mathscr{M}})=\frac{1}{2}\,{\rm{l}}{\rm{n}}\,(det({\bf{K}}+c{\bf{I}}))=\frac{1}{2}\sum _{j=1}^{G}\,{\rm{l}}{\rm{n}}\,({\lambda }_{j}+c)$$

where **I** is the identity matrix. The gradient of the adjusted total correlation dissimilarity measure therefore becomes:

19 $$\frac{\partial {{\mathscr{D}}}_{{\rm{TC}}}}{\partial {\mu }}=\frac{1}{2}\sum _{j=1}^{G}\,\frac{1}{{\lambda }_{j}+c}\frac{\partial {\lambda }_{j}}{\partial {\mu }}=\frac{1}{2}\sum _{j=1}^{G}\,\frac{1}{{\lambda }_{j}+c}({v}_{j}^{{\rm{T}}}\frac{\partial {\bf{K}}}{\partial {\mu }}{v}_{j})$$

To derive an appropriate value for *c*, we make the assumption that the first mode, corresponding to *λ*_1_, accounts for half of the total data variation. Given that the trace of **K** is equal to the sum of its eigenvalues, we can write that ${\rm{tr}}({\bf{K}})={\sum }_{i=1}^{G}{\lambda }_{i}$. In addition, the diagonal elements of the correlation matrix **K** are all equal to 1, which induces that ${\rm{tr}}({\bf{K}})=G={\sum }_{i\mathrm{=1}}^{G}{\lambda }_{i}$. The assumption that the first mode accounts for half of the total data variation therefore yields *λ*_1_ = *G*/2. We then constrain the ratio (*λ*_1_ + *c*)/(*λ*_*G*_ + *c*) to *G*, so that the weights 1/(*λ*_i_ + *c*) in Equation (19) remain within a known, finite range. We also make the assumptions that *c* ≪ *G* and that *λ*_G_ ≪ *c*. This leads to the solution *c* = 0.5. In addition to solving a computational issue, the constant *c* introduces a lower bound on the variance associated with each eigenvector. Initial experiments confirmed that with this choice for *c*, occasional numerical instabilities were successfully eliminated, while not visibly affecting the results in other cases.

Based on Equation (10), the expression of ∂**K**/∂*μ*_*p*_ in Equation (19) becomes:

20 $$\begin{array}{l}\begin{array}{rcl}\frac{\partial {\bf{K}}}{\partial {\mu }_{p}} & = & \frac{\partial }{\partial {\mu }_{p}}(\frac{1}{N-1}{{\rm{\Sigma }}}^{-1}{({\bf{M}}-\overline{{\bf{M}}})}^{{\rm{T}}}({\bf{M}}-\overline{{\bf{M}}}){{\rm{\Sigma }}}^{-1})\\  & = & \frac{1}{N-1}[\frac{\partial {{\rm{\Sigma }}}^{-1}}{\partial {\mu }_{p}}{({\bf{M}}-\overline{{\bf{M}}})}^{{\rm{T}}}({\bf{M}}-\overline{{\bf{M}}}){{\rm{\Sigma }}}^{-1}+{{\rm{\Sigma }}}^{-1}{(\frac{\partial {\bf{M}}}{\partial {\mu }_{p}}-\frac{\partial \overline{{\bf{M}}}}{\partial {\mu }_{p}})}^{{\rm{T}}}({\bf{M}}-\overline{{\bf{M}}}){{\rm{\Sigma }}}^{-1}\\  &  & +\,{{\rm{\Sigma }}}^{-1}{({\bf{M}}-\overline{{\bf{M}}})}^{{\rm{T}}}(\frac{\partial {\bf{M}}}{\partial {\mu }_{p}}-\frac{\partial \overline{{\bf{M}}}}{\partial {\mu }_{p}}){{\rm{\Sigma }}}^{-1}+{{\rm{\Sigma }}}^{-1}{({\bf{M}}-\overline{{\bf{M}}})}^{{\rm{T}}}({\bf{M}}-\overline{{\bf{M}}})\frac{\partial {{\rm{\Sigma }}}^{-1}}{\partial {\mu }_{p}}]\end{array}\end{array}$$

The property of commutativity of the dot product yields:

21 $${v}^{{\rm{T}}}{\bf{AB}}v={v}^{{\rm{T}}}{{\bf{B}}}^{{\rm{T}}}{{\bf{A}}}^{{\rm{T}}}v$$

with A and B, two matrices and *v* a vector. Using Equations (19–21), the derivative of ${{\mathscr{D}}}_{{\rm{TC}}}$ with respect to an element *μ*_*p*_ becomes:

22 $$\begin{array}{c}\frac{\partial {{\mathscr{D}}}_{{\rm{TC}}}}{\partial {\mu }_{p}}=\frac{1}{N-1}\sum _{j=1}^{G}\,[\frac{1}{{\lambda }_{j}+c}\times \{{{\bf{v}}}_{j}^{T}{{\rm{\Sigma }}}^{-1}{({\bf{M}}-\overline{{\bf{M}}})}^{{\rm{T}}}({\bf{M}}-\overline{{\bf{M}}})\\ \,\,\,\,\times \frac{\partial {{\rm{\Sigma }}}^{-1}}{\partial {\mu }_{p}}{{\bf{v}}}_{j}+{{\bf{v}}}_{j}^{T}{{\rm{\Sigma }}}^{-1}{({\bf{M}}-\overline{{\bf{M}}})}^{{\rm{T}}}(\frac{\partial {\bf{M}}}{\partial {\mu }_{p}}-\frac{\partial \overline{{\bf{M}}}}{\partial {\mu }_{p}}){{\rm{\Sigma }}}^{-1}{{\rm{v}}}_{j}\}]\end{array}$$

To obtain ∂Σ^−1^/∂*μ*_*p*_, the diagonal elements ${{\rm{\Sigma }}}_{gg}^{-1}$ of the diagonal matrix Σ^−1^ can be derived one by one:

23 $$\begin{array}{l}\begin{array}{l}\frac{\partial {{\rm{\Sigma }}}_{gg}^{-1}}{\partial {\mu }_{p}}=\frac{\partial }{\partial {\mu }_{p}}{(\frac{1}{N-1}\sum _{i=1}^{N}{({M}_{g,i}-{\overline{M}}_{g})}^{2})}^{-\frac{1}{2}}=-\,\frac{{{\rm{\Sigma }}}_{gg}^{-3}}{N-1}{[{({\bf{M}}-\overline{{\bf{M}}})}^{{\rm{T}}}(\frac{\partial {\bf{M}}}{\partial {\mu }_{p}}-\frac{\partial \overline{{\bf{M}}}}{\partial {\mu }_{p}})]}_{gg}\end{array}\end{array}$$

The quantity ∂**M**/∂*μ*_*p*_ is computed as follows:

24 $$\frac{\partial {M}_{g}({{\bf{T}}}_{g}({\bf{x}},\,{\mu }_{g}))}{\partial {\mu }_{p}}={(\frac{\partial {M}_{g}}{\partial {\bf{x}}})}_{{{\bf{T}}}_{g}({\bf{x}},{{\boldsymbol{\mu }}}_{g})}^{{\rm{T}}}{(\frac{\partial {{\boldsymbol{T}}}_{g}}{\partial {\mu }_{p}})}_{({\bf{x}},{{\boldsymbol{\mu }}}_{g})}$$

It was verified that the derivative $\partial \overline{{\bf{M}}}/\partial {\mu }_{p}$ of the mean intensities was negligibly small and it was therefore ignored in the implementation.

### Related groupwise dissimilarity measures

Huizinga *et al*.^11^ previously presented two dissimilarity measures, the expressions of which are close to the total correlation measure presented in the current article. Huizinga’s dissimilarity measures are based on PCA and rely on the idea that an aligned set of multi-parametric images can be described by a small number of high eigenvalues, since the underlying model *m*_*g*_ is low-dimensional (i.e. the size Γ of ***θ*** is lower than *G*). A misaligned set of multi-parametric images would, on the contrary, be characterized by an eigenvalue spectrum that is more flat: more eigenvalues of average intensity are required for describing the data in that case.

The first dissimilarity measure introduced by Huizinga *et al*.^11^, denoted ${{\mathscr{D}}}_{{\rm{PCA}}}$, quantifies the difference between the sum of all eigenvalues and the sum of the first few eigenvalues:

25 $${{\mathscr{D}}}_{{\rm{PCA}}}( {\mathcal M} )=\sum _{j=1}^{G}\,{\lambda }_{j}-\sum _{j=1}^{L}\,{\lambda }_{j}\,=\,\sum _{j=L+1}^{G}\,{\lambda }_{j}$$

with *L* a user-defined constant with 1≤*L*≤*G*, and ${\sum }_{j=1}^{G}{\lambda }_{j}=tr({\bf{K}})=G$. This means that ${{\mathscr{D}}}_{{\rm{PCA}}}$ is the sum of the lowest *G*−*L* eigenvalues. Contrary to ${{\mathscr{D}}}_{{\rm{PCA}}}$, the second dissimilarity measure, denoted ${{\mathscr{D}}}_{{\rm{PCA2}}}$, does not require the selection of an arbitrary cut-off *L*. It consists of weighting the last eigenvalues more than the first ones:

26 $${{\mathscr{D}}}_{{\rm{PCA2}}}( {\mathcal M} )=\sum _{j=1}^{G}\,j{\lambda }_{j}$$

The dissimilarity measures of Huizinga *et al*.^11^ were developed based on different ideas than total correlation: ${{\mathscr{D}}}_{{\rm{PCA}}}$ and ${{\mathscr{D}}}_{{\rm{PCA2}}}$ were developed based on the concepts of PCA, while ${{\mathscr{D}}}_{{\rm{TC}}}$ is a multivariate derivation of mutual information. Nevertheless, the expressions of ${{\mathscr{D}}}_{{\rm{PCA}}}$ and ${{\mathscr{D}}}_{{\rm{PCA2}}}$, on the one hand, and of ${{\mathscr{D}}}_{{\rm{TC}}}$, on the other hand, happen to resemble each other quite closely: all of them consists of a sum of functions of the eigenvalues.

The main disadvantage of Huizinga’s ${{\mathscr{D}}}_{{\rm{PCA}}}$ with respect to the other techniques is that it requires to choose the cut-off *L*. In ${{\mathscr{D}}}_{{\rm{PCA2}}}$, this user-defined constant is avoided, but the weights *j* in Equation (12) are actually still chosen arbitrarily. For the total correlation dissimilarity measure ${{\mathscr{D}}}_{{\rm{TC}}}$ that we propose is that the contribution of each eigenvalue follows naturally from the derivation of mutual information. A key asset of ${{\mathscr{D}}}_{{\rm{TC}}}$ is therefore that the influence of each eigenvalue is automatically calibrated, because the expression of the dissimilarity measure is derived from the concept of mutual information.

### Implementation codes

The implementation of total correlation will be made available within the open source image registration package elastix, downloadable at the following address: http://elastix.isi.uu.nl.

## Experiments

The quantitative imaging datasets previously considered by Huizinga *et al*.^11^ are covered by the more generic term of multi-parametric datasets, i.e. datasets {*M*_1_, ..., *M*_*G*_} for which the images *M*_*g*_ are characterized by an underlying model *m*_*g*_ describing their intensity values, such that:

27 $${M}_{g}({\bf{x}})={m}_{g}(\theta ({\bf{x}}))+\varepsilon ({\bf{x}})$$

with *θ* a vector (dimension Γ < *G*) containing the model parameters, and *ε* the noise at coordinate **x**. An example of model is the monoexponential model ${m}_{g}(\theta )={S}_{0}\,\exp \,(\,-\,{b}_{g}{u}_{g}^{{\rm{T}}}{\bf{D}}{u}_{g})$ used in diffusion tensor imaging, with *θ* = (*S*_0_, *D*_11_, *D*_12_, *D*_13_, *D*_22_, *D*_23_, *D*_33_), *u*_*g*_ the diffusion gradient direction vector, **D** a 3 × 3 symmetric diffusion tensor, and *b* the b-value^32^.

In particular, Huizinga *et al*.^11^ applied the groupwise dissimilarity measures ${{\mathscr{D}}}_{{\rm{PCA}}}$ and ${{\mathscr{D}}}_{{\rm{PCA2}}}$ to a variety of multi-parametric datasets, and compared the results with other state-of-the-art techniques: pairwise mutual information ${{\mathscr{D}}}_{{\rm{MI}}}$, the accumulated pairwise estimates (APE) introduced by Wachinger and Navab^33^, the groupwise sum of variances designed by Metz *et al*.^9^, and the groupwise mutual information method of Bhatia *et al*.^10^. Huizinga *et al*.^11^ concluded that their measures ${{\mathscr{D}}}_{{\rm{PCA}}}$ and ${{\mathscr{D}}}_{{\rm{PCA2}}}$ yielded better or equal registration results with respect to the other tested methods.

The present experiment uses total correlation ${{\mathscr{D}}}_{{\rm{TC}}}$ as groupwise dissimilarity measure for the registration of the same datasets as in Huizinga *et al*.^11^. On these datasets, the methods of Huizinga *et al*.^11^ were shown to be the best ones, which is why we will compare the registration results of ${{\mathscr{D}}}_{{\rm{TC}}}$ with ${{\mathscr{D}}}_{{\rm{PCA}}}$ and ${{\mathscr{D}}}_{{\rm{PCA2}}}$ only. The results reported by Huizinga *et al*.^11^ for the other dissimilarity measures are directly comparable with the results reported in the present study.

### Description of the six image datasets

The first dataset, denoted CT-LUNG^34^, consists of ten patient subsets containing *G* = 10 three-dimensional CT images of the thorax. The intensity distribution in this dynamic imaging dataset are analogous in all images, which means that the model *m*_*g*_ can be considered as a constant (see Equation (27)): it is therefore a particular case of multi-parametric dataset. The second study, denoted T1MOLLI-HEART^35^, consists of nine *T*_1_-weighted MRI datasets of porcine hearts with transmural myocardial infarction of the lateral wall. *G* = 11 two-dimensional images were acquired for nine subjects. For each registration case, a voxelwise curve fitting was applied to the registered images, producing quantitative *T*_1_ maps. The third study, denoted T1VFA-CAROTID^36^, involves MRIs of the carotid arteries. *G* = 5 three-dimensional images were acquired for 8 human patients. For each patient, the images were registered and fitted to obtain quantitative *T*_1_ maps. The fourth study consists of DW-MR images of the abdominal region, and is denoted ADC-ABDOMEN^12^. Five datasets, each of them including *G* = 19 three-dimensional images, were registered and fitted to produce ADC maps. The fifth study is denoted DTI-BRAIN^37–41^ and consists, for each of the five considered datasets, of registering diffusion tensor images (DTI) of the brain. The number of images to register varied between *G* = 33 and *G* = 70 for each dataset^11^. The fitted parameter is the mean diffusivity (*MD*). The sixth study involves DCE images of the abdomen. Five DCE-ABDOMEN^42^ datasets were acquired, each of them containing *G* = 160 three-dimensional images. The fitted parameter of interest considered in this study is *K*^*trans*^. The full descriptions of the fitting models are provided by Huizinga *et al*.^11^.

All human data used in this study came from anonymized datasets. Data from the CT-LUNG dataset was obtained from a publicly available dataset^34^ available at the following address: https://www.dir-lab.com. The ethics committee of the Academisch Medisch Centrum, Amsterdam, the Netherlands, approved the research related to the T1VFA-CAROTID and DCE-ABDOMEN datasets. The Research Ethics Committee of the Royal Marsden Hospital, United Kingdom, approved the research related to the ADC-ABDOMEN dataset. The medical ethics committee for research in humans of the University Medical Center Utrecht, the Netherlands, approved the research performed on the DTI-BRAIN dataset. Informed consent was obtained from all patients in human datasets. Porcine data from the T1MOLLI-HEART dataset were approved by the Animal Ethics Committee of the Erasmus MC Rotterdam, the Netherlands. All studies were carried out in accordance with the relevant guidelines and regulations.

### Registration characteristics

We selected the same registration settings as Huizinga *et al*.^11^, for comparison purposes. The dissimilarity measures were applied in identical environments. Apart from the dissimilarity measure, all other registration settings such as the choice of optimizer, number of resolutions, number of iterations or number of considered samples, were identical. Two resolutions of 1000 iterations were used for all six image datasets. To account for deformations caused by heart-pulsations and breathing, we used a B-spline transformation model for the CT-LUNG, T1MOLLI-HEART, T1VFA-CAROTID, ADC-ABDOMEN and DCE-ABDOMEN datasets. The registrations were performed for three distinct B-spline grid spacings: 32 mm, 64 mm and 128 mm for the T1MOLLI-HEART, ADC-ABDOMEN, DCE-ABDOMEN datasets, 8 mm, 16 mm and 32 mm for the T1VFA-CAROTID dataset, and 6 mm, 13 mm and 20 mm for the CT-LUNG dataset. All results are reported as supplementary material (Tables S1 to S6). Results for the intermediate values of the spacings (i.e. either 64 mm, 16 mm or 13 mm), are reported in the Results section of this article. To account for deformations caused by head motion and eddy current distortions, we used an affine transformation model for the DTI-BRAIN dataset. When applying ${{\mathscr{D}}}_{{\rm{PCA}}}$, the value of *L* was 1 for CT-LUNG, 3 for T1MOLLI-HEART, 1 for T1VFA-CAROTID, 4 for ADC-ABDOMEN, 7 for DTI-BRAIN, and 4 for DCE-ABDOMEN.

### Evaluation measures

No ground truth alignment was available for any of the six datasets considered. However, registration performance was evaluated based on four different measures, described in Huizinga *et al*.^11^, and briefly described in this section.

The first two measures are based on landmark correspondence and overlap of volumes of interest. Landmarks were manually defined on images of the T1VFA-CAROTID and DCE-ABDOMEN datasets. The correspondence between the corresponding landmarks was evaluated by computing a mean target registration error (mTRE). In the T1MOLLI-HEART case, segmentations of the myocardium were outlined on between 6 and 9 images per patient. In the ADC-ABDOMEN case, the spleen was manually delineated on 8 images. For these two cases, the overlap between the segmented structures was then evaluated using a Dice coefficient. For the DTI-BRAIN study, neither landmarks nor structures could be reliably identified on the diffusion weighted images, which is why no overlap or point correspondence was calculated^11^.

The second measure quantifies the smoothness of the transformation obtained through registration. Extreme and non-smooth deformations are unexpected. The smoothness of the deformation field can therefore be used to identify such undesirable transformations. A smoothness quantification can be obtained by computing the standard deviation of the determinant of ∂***T***_*g*_/∂**x** over all **x** for all images: ${{\rm{STD}}}_{{\rm{\det }}(\partial {{\boldsymbol{T}}}_{g}/\partial {\bf{x}})}$. Smoothness was quantified for all datasets except for DTI-BRAIN because an affine transformation was used in that last case. The smoother the transformation, the lower the quantity ${{\rm{STD}}}_{{\rm{\det }}(\partial {{\boldsymbol{T}}}_{g}/\partial {\bf{x}})}$.

The last evaluation measure is an uncertainty estimation of the qMRI fit. For the five qMRI datasets, curve fittings were performed to generate quantitative images. The fitted values were evaluated in the myocardium for the T1MOLLI-HEART dataset (*T*_1_ values), in the carotid artery wall for the T1VFA-CAROTID dataset (*T*_1_ values), in the spleen for the ADC-ABDOMEN dataset (ADC values), in the brain parenchyma for the DTI-BRAIN dataset (MD values), and in the pancreas for the DCE-ABDOMEN dataset (*K*^*trans*^ values). The qMRI models were fitted using a maximum likelihood estimator that takes into account the Rician characteristic of the noise in MR data. We used the fitting same method as Huizinga *et al*.^11^, based on the work of Poot *et al*.^43^. The uncertainty of these fitted qMRI model parameters can be quantified by the 90^th^ percentile of the square root of Cramér-Rao lower bound (CRLB), which provides a lower bound for the variance of the maximum likelihood parameters. This uncertainty estimate is denoted 90^th^ $\sqrt{{\rm{CRLB}}}$.

### Assessment of multivariate joint normality

As mentioned in the Method section, the computation of the total correlation dissimilarity measure ${{\mathscr{D}}}_{{\rm{TC}}}$ that we propose is based on the approximation that the intensity distribution of the images to register is multivariate normal. For most datasets, however, the intensity distribution is expected not to be multivariate normal. The underlying idea is that the approximated dissimilarity measure will result in the same minimization result as if the approximation had not been done.

A second interest of the experimental setting is therefore to evaluate how multivariate normal the intensity distributions are for the six types of datasets that are registered in this study, and in the light of the registration accuracy results, to assess whether the approximation that we made can be considered as sensible on multi-parametric datasets.

The joint normality of two images can be assessed by computing and visualizing their joint histogram. Assessing joint normality on more images requires other methods. A possible graphical approach to analyze the multivariate joint normality of *G* images is to compare the distributions of observed Mahalanobis distances with the distribution of a chi-square distribution with *G* degrees of freedom ${\chi }_{G}^{2}$. A squared Mahalanobis distance ${d}_{i}^{2}$ (with *i* = 1...*N*) can be computed at each voxel location *M*_*g*_(*i*), by: ${d}_{i}^{2}=({y}_{i}-{y}_{m}{)}^{T}{{\bf{S}}}^{-1}({y}_{i}-{y}_{m})$, with *y*_*i*_ = [*M*_1_(*i*), .., *M*_*G*_(*i*)]^*T*^, the sample mean vector ${y}_{m}={\sum }_{i\mathrm{=1}}^{N}{y}_{i}/N$, and the sample covariance ${\bf{S}}={\sum }_{i\mathrm{=1}}^{N}({y}_{i}-{y}_{m}){({y}_{i}-{y}_{m})}^{T}/(N-\mathrm{1)}$. It has been shown that the sample squared Mahalanobis distance converges to ${\chi }_{G}^{2}$ when ${y}_{i} \sim {{\mathscr{N}}}_{k}({y}_{m},\,{\bf{S}})$^44^. To graphically check whether the distribution of intensities of **M** is joint normal, we will plot the cumulative distribution function (CDF) of *d*^2^ and ${\chi }_{G}^{2}$ in the same graph. If the CDF of the squared Mahalanobis distances *d*^2^ approaches this of ${\chi }_{G}^{2}$, then we will consider the data as joint normal.

### Computational efficiency of total correlation ${{\mathscr{D}}}_{{\rm{TC}}}$

To study the computational efficiency of the proposed total correlation dissimilarity measure ${{\mathscr{D}}}_{{\rm{TC}}}$, the average time per iteration is studied by varying three registration parameters: the number of images *G* that are simultaneously registered, the number of spatial samples taken to evaluate the groupwise dissimilarity measure, and the number of B-spline control points of the transformation model used to warp the images. The influence of these three parameters on the average time per iteration is studied by varying each of them while setting the two remaining ones at values in the range of those described in the Registration characteristics section:

- when the number of B-spline control points evolves, the number of images *G* is set to 50, and the number of spatial samples to 1024. The numbers of B-spline control points per image vary between 50 and 20000;
- when the number of images *G* evolves, the number of B-spline control points is set to 500 per image, and the number of spatial samples to 1024. The numbers of images *G* cover the characteristics of the images described in the ‘Description of the six image datasets’ section (i.e. *G* = 5...160);
- when the number of spatial samples evolves, the number of B-spline control points is set to 500 per image, and the number of images *G* is set to 50. We considered numbers of spatial samples between 16 samples and 8192.

## Electronic supplementary material

Supplementary information
