# Supplementary material for: Groupwise image registration based on a total correlation dissimilarity measure for quantitative MRI and dynamic imaging data
Source: Sci Rep. 2018 Aug 30;8:13112. doi: 10.1038/s41598-018-31474-7 (PMC6117310; doi:10.1038/s41598-018-31474-7)
Supplement: Supplementary file 1 — Supplementary information [file 41598_2018_31474_MOESM1_ESM.pdf]

Supplementary information

Groupwise image registration based on a total correlation dissimilarity measure for quantitative MRI and dynamic imaging data

Jean-Marie Guyader, Wyke Huizinga, Dirk H. J. Poot, Matthijs van Kranenburg, André Uitterdijk, Wiro J. Niessen, Stefan Klein

Cells with grey background are already included in the main article.

Table S1: T1MOLLI-HEART. Mean and standard deviation over all subjects for all evaluation measures and control point spacings 32 mm, 64 mm and 128 mm, and for reference images 1, 4, 7 and 11.

(a) Dice [%]

	32				64				128			
	1	4	7	11	1	4	7	11	1	4	7	11
Mis.	48 ± 8	48 ± 8	48 ± 8	48 ± 8	48 ± 8	48 ± 8	48 ± 8	48 ± 8	48 ± 8	48 ± 8	48 ± 8	48 ± 8
$\mathcal{D}_{MI}$	26 ± 11	22 ± 10	22 ± 15	39 ± 14	37 ± 11	34 ± 16	19 ± 15	52 ± 7	44 ± 14	40 ± 13	24 ± 17	51 ± 7
$\mathcal{D}_{PCA}$	54 ± 9	54 ± 9	54 ± 9	54 ± 9	53 ± 7	53 ± 7	53 ± 7	53 ± 8	51 ± 9	50 ± 9	51 ± 9	51 ± 9
$\mathcal{D}_{PCA2}$	52 ± 13	52 ± 13	52 ± 13	52 ± 13	52 ± 11	52 ± 10	52 ± 11	53 ± 10	52 ± 7	52 ± 8	52 ± 8	52 ± 8
$\mathcal{D}_{TC}$	52 ± 13	52 ± 12	52 ± 13	53 ± 13	53 ± 11	53 ± 11	53 ± 11	53 ± 11	53 ± 7	53 ± 8	54 ± 7	53 ± 8

(b) $\mathrm{STD}_{|\partial \mathbf{T}g/\partial \mathbf{x}|}$ [%]

	32				64				128			
	1	4	7	11	1	4	7	11	1	4	7	11
Mis.	0 ± 0	0 ± 0	0 ± 0	0 ± 0	0 ± 0	0 ± 0	0 ± 0	0 ± 0	0 ± 0	0 ± 0	0 ± 0	0 ± 0
$\mathcal{D}_{MI}$	24 ± 8	28 ± 8	29 ± 9	10 ± 4	7 ± 2	7 ± 2	8 ± 3	3 ± 1	1 ± 0	1 ± 0	1 ± 0	1 ± 0
$\mathcal{D}_{PCA}$	6 ± 4	4 ± 2	4 ± 2	4 ± 1	2 ± 1	1 ± 1	2 ± 1	1 ± 1	0 ± 0	0 ± 0	0 ± 0	0 ± 0
$\mathcal{D}_{PCA2}$	6 ± 2	5 ± 2	6 ± 2	4 ± 1	1 ± 1	1 ± 1	2 ± 1	1 ± 1	0 ± 0	0 ± 0	0 ± 0	0 ± 0
$\mathcal{D}_{TC}$	7 ± 3	6 ± 3	7 ± 3	5 ± 1	2 ± 1	2 ± 1	2 ± 1	1 ± 1	0 ± 0	0 ± 0	0 ± 0	0 ± 0

(c) 90% $\sqrt{\mathrm{CRLB}}$ T_1 [ms]

	32				64				128			
	1	4	7	11	1	4	7	11	1	4	7	11
Mis.	92 ± 19	92 ± 19	92± 19	92 ± 19	92 ± 19	92 ± 19	92 ± 19	92 ± 19	92 ± 19	92 ± 19	92 ± 19	92 ± 19
$\mathcal{D}_{MI}$	119 ± 26	157 ± 61	467±610	93 ± 19	97 ± 16	103 ± 18	146 ± 52	83 ± 13	91 ± 14	95 ± 16	121 ± 29	81 ± 12
$\mathcal{D}_{PCA}$	83 ± 17	79 ± 17	78± 11	85 ± 16	87 ± 16	84 ± 20	83 ± 13	85 ± 13	88 ± 14	89 ± 21	103 ± 50	85 ± 10
$\mathcal{D}_{PCA2}$	81 ± 15	79 ± 16	79± 12	79 ± 12	83 ± 12	79 ± 14	80 ± 10	81 ± 10	84 ± 12	82 ± 15	82 ± 10	81 ± 11
$\mathcal{D}_{TC}$	77 ± 14	75 ± 11	73± 14	74 ± 12	77 ± 13	75 ± 11	73 ± 13	74 ± 11	79 ± 12	76 ± 11	76 ± 13	75 ± 11

Table S2: T1VFA-CAROTID. Means and standard deviation over all subjects for all evaluation measures and control point spacings 8 mm, 16 mm and 32 mm.

(a) mTRE [mm]

	8	16	32
Mis.	1.47±0.54	1.47±0.54	1.47±0.54
$\mathcal{D}_{MI}$	1.26±0.44	1.22±0.43	1.23±0.45
$\mathcal{D}_{PCA}$	1.25±0.56	1.11±0.42	1.10±0.43
$\mathcal{D}_{PCA2}$	1.13±0.46	1.08±0.39	1.10±0.43
$\mathcal{D}_{TC}$	1.19±0.50	1.09±0.40	1.10±0.42

(b) $\mathrm{STD}_{|\partial \mathbf{T}g/\partial \mathbf{x}|}$ [%]

	8	16	32
Mis.	0 ± 0	0 ± 0	0 ± 0
$\mathcal{D}_{MI}$	7 ± 1	2 ± 0	0 ± 0
$\mathcal{D}_{PCA}$	6 ± 1	2 ± 1	0 ± 0
$\mathcal{D}_{PCA2}$	5 ± 1	1 ± 0	0 ± 0
$\mathcal{D}_{TC}$	6 ± 1	1 ± 0	0 ± 0

(c) 90% $\sqrt{\mathrm{CRLB}}$ T_1 [ms]

	8	16	32
Mis.	> 1000	> 1000	> 1000
$\mathcal{D}_{MI}$	530 ± 136	501 ± 83	523 ± 93
$\mathcal{D}_{PCA}$	540 ± 154	498 ± 93	530 ± 94
$\mathcal{D}_{PCA2}$	532 ± 154	510 ± 110	523 ± 87
$\mathcal{D}_{TC}$	533 ± 150	500 ± 96	528 ± 96

Table S3: ADC-ABDOMEN. Mean and standard deviation over all subjects for all evaluation measures and control point spacings of 32 mm, 64 mm and 128 mm.

(a) Dice [%]

	32	64	128
Mis.	70 ± 4	70 ± 4	70 ± 4
$\mathcal{D}_{MI}$	61 ± 18	64 ± 16	73 ± 8
$\mathcal{D}_{PCA}$	65 ± 13	71 ± 5	71 ± 4
$\mathcal{D}_{PCA2}$	75 ± 7	75 ± 5	73 ± 5
$\mathcal{D}_{TC}$	72 ± 6	74 ± 5	73 ± 5

(b) $\mathrm{STD}_{|\partial \mathbf{T}g/\partial \mathbf{x}|}$ [%]

	32	64	128
Mis.	0 ± 0	0 ± 0	0 ± 0
$\mathcal{D}_{MI}$	25 ± 11	8 ± 3	2 ± 1
$\mathcal{D}_{PCA}$	11 ± 3	3 ± 1	1 ± 0
$\mathcal{D}_{PCA2}$	8 ± 2	3 ± 1	0 ± 0
$\mathcal{D}_{TC}$	16 ± 5	5 ± 2	1 ± 0

(c) 90% $\sqrt{\mathrm{CRLB}}$ ADC [μm^2/ms]

	32	64	128
Mis.	0.24±0.06	0.25±0.05	0.29±0.05
$\mathcal{D}_{MI}$	0.20±0.08	0.23±0.10	0.35±0.16
$\mathcal{D}_{PCA}$	0.15±0.04	0.23±0.06	0.46±0.27
$\mathcal{D}_{PCA2}$	0.23±0.03	0.27±0.05	0.41±0.18
$\mathcal{D}_{TC}$	0.33±0.05	0.32±0.05	0.38±0.14

Table S4: DCE-ABDOMEN. Mean and standard deviation over all subjects for all evaluation measures and control point spacings 32 mm, 64 mm and 128 mm.

(a) mTRE [mm]

	32	64	128
Mis.	8.49±4.54	8.49±4.54	8.49±4.54
$\mathcal{D}_{MI}$	6.73±2.02	6.46±2.32	6.47±2.37
$\mathcal{D}_{PCA}$	6.21±2.25	6.11±2.32	6.24±2.37
$\mathcal{D}_{PCA2}$	5.89±2.23	5.99±2.17	6.18±2.27
$\mathcal{D}_{TC}$	7.01±1.82	6.18±2.40	6.23±2.30

(b) $\mathrm{STD}_{|\partial \mathbf{T}g/\partial \mathbf{x}|}$ [%]

	32	64	128
Mis.	0 ± 0	0 ± 0	0 ± 0
$\mathcal{D}_{MI}$	20 ± 9	4 ± 2	1 ± 1
$\mathcal{D}_{PCA}$	11 ± 4	4 ± 2	1 ± 0
$\mathcal{D}_{PCA2}$	6 ± 3	2 ± 1	0 ± 0
$\mathcal{D}_{TC}$	11 ± 4	4 ± 2	1 ± 0

(c) 90% $\sqrt{\mathrm{CRLB}}$ K^{trans} [min^{-1}]

	32	64	128
Mis.	2.84±2.30	2.84±2.30	2.84±2.30
$\mathcal{D}_{MI}$	3.85±2.41	3.64±4.13	2.54±2.58
$\mathcal{D}_{PCA}$	1.69±1.48	1.52±1.18	1.46±1.09
$\mathcal{D}_{PCA2}$	1.17±0.87	1.27±0.92	1.38±1.16
$\mathcal{D}_{TC}$	1.78±1.74	1.87±1.81	1.54±1.38

Table S5: DTI-BRAIN. Mean and standard deviation of the 90% $\sqrt{\mathrm{CRLB}}$ MD [μm^2/ms] over all subjects.

	Affine
Mis.	0.096 ± 0.029
$\mathcal{D}_{MI}$	0.084 ± 0.028
$\mathcal{D}_{PCA}$	0.085 ± 0.029
$\mathcal{D}_{PCA2}$	0.084 ± 0.028
$\mathcal{D}_{TC}$	0.085 ± 0.029

Table S6: CT-LUNG. Mean and standard deviation over all subjects for all evaluation measures and control point spacings 6 mm, 13 mm and 20 mm.

(a) mTRE [mm]

	6	13	20
Mis.	6.72±2.51	6.72±2.51	6.72±2.51
$\mathcal{D}_{MI}$	1.78±0.40	1.43±0.23	1.45±0.21
$\mathcal{D}_{PCA}$	1.47±0.60	1.40±0.37	1.45±0.34
$\mathcal{D}_{PCA2}$	1.72±0.78	1.56±0.55	1.59±0.49
$\mathcal{D}_{TC}$	1.48±0.62	1.42±0.40	1.47±0.37

(b) $\mathrm{STD}_{|\partial \mathbf{T}g/\partial \mathbf{x}|}$ [%]

	6	13	20
Mis.	0 ± 0	0 ± 0	0 ± 0
$\mathcal{D}_{MI}$	28 ± 8	15 ± 4	11 ± 3
$\mathcal{D}_{PCA}$	12 ± 3	8 ± 2	7 ± 2
$\mathcal{D}_{PCA2}$	10 ± 2	7 ± 2	6 ± 1
$\mathcal{D}_{TC}$	12 ± 3	8 ± 2	7 ± 2
